# Miscellaneous Notices

**Published:** 1857-01

**Authors:** 


### MISCELLANEOUS NOTICES.

*College of Dentists, England.*—In noticing the advent of the British Journal of Dental Science in the last number of our quarterly, we took occasion to direct attention to one of the editorials which announced the fact that an effort was being made, by our transatlantic brethren of England, to organize a Dental Institute or Society. Since the publication of that issue, we received a letter from a professional friend of London, informing us that one or two preliminary meetings of a number of dentists of that metropolis, with a view to such organization, had been held, and from the unanimity of feeling which prevailed, a favorable result was confidently anticipated. Subsequently to the receipt of this letter, we have received a number of the London Observer, containing a communication purporting to give a history of the origin and progress of the movement. From this, we learn, that a general meeting was held on Tuesday evening, December the 16th, and that the prominent members of the profession in attendance, from confidence in themselves as well as the justice of the cause in which they were engaged, entertained but little doubt as to the result.

It seems from this communication, that some eighteen dentists, members of the College of Surgeons, secretly memorialized their college to grant diplomas without consulting the whole body of the dental profession. In consequence of this, a number of the leading dentists, at once put on foot, a movement which has resulted in the present organization, which opposes any interference of the College of Surgeons in the dental specialty of medicine, on the ground that men not educated for the practice, cannot judge of the qualifications necessary to a calling so distinct from their own, and of the practical details of which they are wholly ignorant.

The authorities of the college, as appears from the correspondent of the Observer, have not, to the present time, responded to the memorialists, owing, as is believed by the author of the communication, to the secret manner in which they were approached as well as to the obvious injury intended to their professional brethren.

The author of the communication in the Observer, copies from the Medical Circular, the following letter, written, as he says, by a member of the College of Surgeons. The writer, in alluding to the memorialists, says :

"In accordance with the *general feeling of the profession that a Society of Dentists should be formed;* for which purpose a meeting was convened in November, 1855, and at which meeting it was decided to memorialise the College of Surgeons. Now, I ask any one of ordinary capacity, whether on seeing the above statement, the existence of a *general feeling* in a large body of men, urged as a plea for pressing the subject on the notice of the authorities, would not suppose that the body itself, or its delegates, had been *called together* to give it utterance? Of course he would. No such thing; on the contrary, the body, with its general feeling, knew nothing of what some of the fingers and toes had done until many months after the memorial had been presented; that they had no share in getting it up will be clear to any one who will take the trouble to read the list of eighteen names appended to it, and unless the said eighteen embody in themselves the *general feeling* of the profession, the pretence set forth in the prospectus is a false one. We are further informed on the one hand that the memorial presented to the College twelve months since, is still under the consideration of the council, and on the other it is asserted that the College at once told the memorialists that their charter gave them no power to comply with the request; but if the College had the power it would do so. Would it? Then why has it remained twelve months under consideration? Many hard things are said of the College, and, perhaps, she has not been a kind mother to all her progeny; but I can never think so badly of her as to believe that while in all other departments the standard of qualification is being raised, she would take a step, or rather a stride, backward, to suit the convenience of *individuals,* and to perpetuate an evil which ought to be remedied at the very earliest moment."

Judging from the Observer's correspondent, the members of the profession who have attended these preliminary meetings, are of the opinion, that they can furnish a more competent board of examiners from among themselves, being an independent body of practitioners, than the College. The writer, from whom we have gathered the foregoing facts, commends the course of the committee to whom the arrangement of the plan of the new College or Society has been entrusted, in holding their meetings publicly. We also learn that at a public meeting recently held at Cheltenham, the entire body of dentists and their assistants, who, as appears, were admitted, with a single exception, sent in their adhesion to the proposed college. It will receive the support, we learn, from the dentists of all the provincial towns of England.

The proceedings of the public meetings, together with the general laws adopted, it is said, will soon be made known to the profession. We trust that some one of our professional friends of London, will furnish us with a full and detailed description of the above movement, together with the precise object intended to be accomplished by it. Every effort to advance dental science and elevate the dignity of the profession, should receive the cordial and active support of all its members. We are glad that our trans-atlantic brethren are waking up to the importance of association and union of effort for the promotion of these great objects.

---

*Polishing Stones.*—The value of Arkansas rock, ground into thin pieces, for removing the file scratches from the surfaces of teeth after filing, and the surfaces of gold fillings preparatory to burnishing or polishing, is known to very many members of the dental profession. It has been used for this purpose by many practitionres for some twelve or thirteen years, but as the surface of this particular variety of rock, when used for grinding down a filling, soon fills with gold, it becomes necessary to rub it frequently on a corundum or emery slab or pumice-stone to restore the efficiency of its action on a metallic substance. But this inconvenience is not experienced in the use of every variety of rock having a fine sharp grit. The senior editor recently received from the New York Teeth Manufacturing Company, through their Baltimore agent, Mr. Snowden, several specimens of Lake Superior rock, reduced to convenient sizes and forms, which he has found to be wholly free from the above mentioned objection. For operations on the teeth and sharpening excavators and other dental instruments it is certainly vastly ***superior*** to the Arkansas rock. It cuts more rapidly and leaves an equally smooth surface. We, therefore, take pleasure in recommending the use of it to our professional brethren.

---

*North Carolina Dentists Society.*—It will be seen from the Proceedings published in another part of the present No. of the Journal, that the alumni of dental colleges practicing in the State of North Carolina, have united in the organization of a Society with a view to the elevation of the dignity of their profession and the advancement of the science of their calling. We trust their efforts may be crowned with success. Their meetings, we believe, are to be held annually, and these yearly convocations can scarcely fail to prove highly beneficial to all who attend them, and no trifling matter should prevent any one from being present on these occasions. The collective experience and professional knowledge accumulated during a year, of so respectable a body of practitioners, made public at each successive meeting, will certainly richly compensate every one for the time and expense incurred in attending it. That the members may realize great benefit from their organization is earnestly to be desired. We wish them abundant success.

---

*Dr. Paris.*—We copy from the Medical Times and Gazette, of London, the following notice of the death of the distinguished Dr. Paris.

"At the moment of going to press, we hear that Dr. Paris, President of the Royal College of Physicians, died on the morning of the 24th inst. (December.)

"His last literary employment was preparing a new edition of his successful work, 'Philosophy in Sport made Science in Earnest,' the last sheets of which he revised only a few days before his death. Want of space obliges us to postpone any further notice of his life and works."

*Index to Volume Sixth.*—The index to the sixth volume of the Journal should have appeared in the last No., but it was forgotten until too late to remedy the omission. We, therefore, send it out in the present No., and if any of our subscribers have had that volume bound the index may be readily inserted.

*Grinding Apparatus.*—One of the most beautiful and ingeniously contrived portable grinding apparatus we have ever seen was presented to the senior editor, a few months since, by Dr. Reynolds, dentist, of Philadelphia. It is both ornamental and useful. It occupies but little space, and can be readily attached to the projecting edge of a table. It carries three wheels, which are rapidly revolved by turning a small crank.

*Pratt's Patent Safety Lamp and Feeder.*—The design of the invention as set forth in a pamphlet before us, is to render safe, under all circumstances, those explosive fluids of various names, which have entered so largely into consumption for purposes of illumination, and which from the scarcity of other articles, and from the clearness and brilliancy of the light produced by them, have become of prime necessity with all classes, who from locality or other causes cannot avail themselves of the use of gas. In view of the many disastrous accidents which have hitherto occurred, we hail with pleasure an invention which seems to place beyond possibility their recurrence. The merits of the invention in question consists in its simplicity and indestructibility, perfectly carrying out in practice a theoretical principle which under all forms of application hitherto attempted, has proved insufficient for the purpose. The orifices of reception and delivery, both of lamp and feeder, are protected by a diaphragm so constructed, that, while within small limits it presents a great amount of conducting surface and material, which at the same time allows of the greatest interspace to replenish the lamp, which may be safely done even while lighted. Indeed we have repeatedly witnessed the filling *of the lamp,* though a flame kindled upon both lamp and feeder, the fluid passing harmlessly through a powerful blaze. The safety principle being inherent and not depending for its efficacy upon care and presence of mind, seems to fulfil all the conditions necessary to safety.

To quote from the circular, "It appears, that step by step, the various causes of danger developed themselves under careful experiment, and in the same gradual manner, have been overcome by devices, which though simple and seemingly trivial in themselves, have in this case made good the old adage, that '*though perfection is no little thing, yet that a little thing may make perfection.*'" We learn from the inventor, that he will apply his safety attachments to the spirit lamps and vessels, so much used in our *mechanical departments, operations and laboratories,* which we hope to present to our readers, with a cut and description, in our next issue. The Company will produce none but reliable articles, and will consult not only the wants of the humblest consumer, but will also minister to the demands of those whose means and tastes require articles of the greatest elegance.

---

BALTIMORE, *Jan. 5th,* 1857.

PROFESSOR HARRIS:

*Dear Sir*—Allow me through your Journal to make public acknowledgement of the following donations to the Baltimore College of Dental Surgery, received within the year past.

From the New York Teeth Manufacturing Company, a square furnace containing one baking and two smaller annealing muffles.

From Dr. A. Merrit Asay, Race street, Philadelphia, a highly finished operating chair.

From Dr. W. H. Helme, of Providence, R. I., one of his dental ratchet lathes.

From Dr. Samuel Mallet, of New Haven, Ct., a self adjusting rivet-hole punch: also two specimens of osseous union of the teeth.

From Dr. D. W. Perkins, Rome, N. Y., one of his patent dental operating chairs.

From Dr. W. F. Bason, of Salsbury, N. C., a case of osseous union between a lateral incisor and a supernumerary cuspidatus.

We are glad to receive such substantial tokens of regard for our Institution, not only as evincing such regard, but because of their intrinsic value to us in our work of practical instruction.

May we gently hint that such generosity is worthy of imitation. It is not every one who could make a donation so expensive as some of the above: but there are very few who might not contribute some specimen of diseased structure or abnormal development. Such specimens, isolated, have comparatively small value; but collected and arranged they become to the teacher invaluable means of instruction.

Respectfully, yours,

P. H. AUSTEN,
*Dean of the Faculty B. C. D. S.*

